# Biliopleural Fistula Following Percutaneous Transhepatic Drainage: A Rare Cause of Bilious Pleural Effusion

**DOI:** 10.7759/cureus.100653

**Published:** 2026-01-02

**Authors:** Rim A Boutari, Abed AlRaouf Kawtharani, Ralph C Harrouk, Ali N Hamade, Leonardo N Naffah, Antoine S Geagea

**Affiliations:** 1 Gastroenterology and Hepatology, Lebanese University Faculty of Medical Sciences, Beirut, LBN; 2 Internal Medicine, Lebanese University Faculty of Medical Sciences, Beirut, LBN; 3 Pulmonology and Critical Care, Lebanese University Faculty of Medical Sciences, Beirut, LBN; 4 Radiology, Lebanese University Faculty of Medical Sciences, Beirut, LBN; 5 Gastroenterology and Hepatology, Lebanese Hospital Geitaoui University Medical Center, Beirut, LBN

**Keywords:** biliopleural fistula, choledocholithiasis, hepatobiliary intervention, percutaneous transhepatic drainage, pleural effusion

## Abstract

Biliopleural fistula is a rare but serious complication of hepatobiliary interventions. It may present with atypical respiratory symptoms and requires a high index of suspicion for diagnosis. We report the case of a 79‑year‑old woman with choledocholithiasis complicated by failed endoscopic retrograde cholangiopancreatography (ERCP), post‑ERCP pancreatitis, and cholangitis. Following percutaneous transhepatic drainage (PTD) with external biliary catheter placement, she developed a loculated right‑sided pleural effusion. Diagnostic thoracentesis revealed bilious pleural fluid, and magnetic resonance cholangiopancreatography (MRCP) confirmed a fistulous tract between the intrahepatic biliary system and the pleural space. Surgical thoracotomy with decortication established the diagnosis and resolved the condition. This case highlights the importance of recognizing biliopleural fistula as a potential complication of PTD. Early imaging and timely surgical intervention are critical to prevent respiratory compromise.

## Introduction

Biliopleural fistula is an uncommon complication of hepatobiliary disease, defined as an aberrant communication between the biliary system and the pleural cavity. Although most documented cases are secondary to hepatic trauma, abscess formation, or neoplastic invasion, iatrogenic causes, particularly following hepatobiliary interventions, are increasingly recognized [1].

Percutaneous transhepatic drainage (PTD) is widely employed when endoscopic retrograde cholangiopancreatography (ERCP) is unsuccessful or contraindicated. Despite its efficacy, PTD carries risks including hemorrhage, infection, and bile leakage [2]. The anatomical proximity of the right hepatic lobe to the diaphragm, coupled with disruption of the hepatic capsule, may facilitate formation of a transdiaphragmatic fistulous tract [3].

Despite its clinical significance, biliopleural fistula remains underreported, with few cases detailing diagnostic pathways, imaging findings, and therapeutic outcomes. Importantly, no systematic incidence has been established for post‑PTD biliopleural fistula, with the literature limited to isolated case reports and small series, underscoring its rarity. The condition poses a diagnostic challenge, particularly in the absence of abdominal symptoms, and requires suspicion in patients with recent biliary interventions who develop unexplained pleural effusions [4].

We present a rare case of biliopleural fistula following PTD in a patient with choledocholithiasis and post‑ERCP pancreatitis. The case is notable for its atypical respiratory presentation, diagnostic complexity, and need for surgical decortication.

## Case presentation

A 79‑year‑old woman with a history of hypertension, dyslipidemia, benign paroxysmal positional vertigo, anemia, and prior cholecystectomy was admitted to an outside hospital with epigastric pain. Magnetic resonance cholangiopancreatography (MRCP) revealed choledocholithiasis with a 15‑mm dilated common bile duct (CBD) containing a 5‑mm obstructing stone. ERCP was attempted but was unsuccessful. She subsequently developed post‑ERCP pancreatitis complicated by cholangitis, requiring intensive care unit admission and empirical treatment with meropenem and teicoplanin. After stabilization, she was transferred to our institution.

On admission, she had persistent jaundice and right upper quadrant tenderness. Laboratory tests showed markedly elevated transaminases (aspartate aminotransferase (AST) 1573 U/L, alanine transaminase (ALT) 1233 U/L), cholestatic enzymes (alkaline phosphatase (ALP) 994 U/L, gamma-glutamyl transferase (GGT) 1024 U/L), total/direct bilirubin 8.2/7.1 mg/dL, lipase 518 U/L, and C‑reactive protein (CRP) 51 mg/L (Table 1).

**Table 1:** Laboratory values at initial admission and re‑presentation, including pleural fluid analysis AST: aspartate aminotransferase; ALT: alanine aminotransferase; GGT: gamma‑glutamyl transferase; CRP: C‑reactive protein

Parameter	First admission	Re-presentation	Reference range
AST (U/L)	1573	–	0–35
ALT (U/L)	1233	29	4–42
Alkaline phosphatase (U/L)	994	271	44–147
GGT (U/L)	1024	266	5–35
Total bilirubin (mg/dL)	8.2	1.2	0.3–1.2
Direct bilirubin (mg/dL)	7.1	1.0	0.0–0.3
Lipase (U/L)	518	186	13-60
CRP (mg/L)	51	190	< 5
Pleural fluid bilirubin (mg/dL)	–	1.251	–
Serum bilirubin at thoracentesis (mg/dL)	–	0.9	0.3–1.2

Computed tomography (CT) of the abdomen and pelvis demonstrated significant intra‑ and extrahepatic biliary dilatation, with a CBD diameter of 25 mm. PTD with external biliary catheter placement was performed, resulting in clinical and biochemical improvement. She was discharged in stable condition.

**Figure 1:**
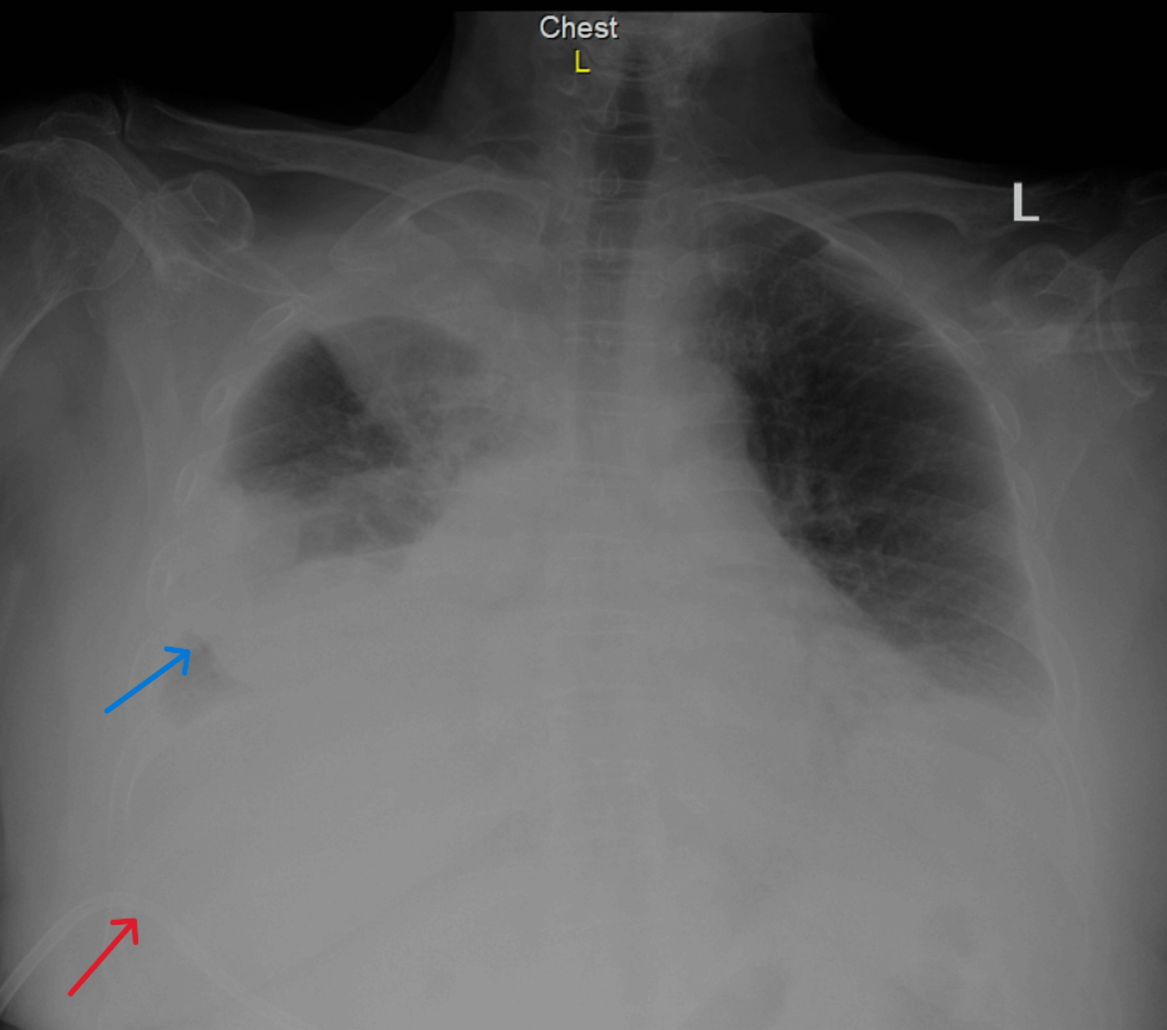
Chest radiograph showing right-sided loculated pleural effusion Blue arrow: right-sided loculated pleural effusion; Red arrow: Percutaneous transhepatic drain

Two weeks later, she re‑presented with pain and erythema at the drain site, fever (maximum 38.5 °C), oxygen desaturation to 86%, and mild bile leakage. At that time, she was on oral amoxicillin‑clavulanate and ciprofloxacin. Examination revealed severe right upper quadrant tenderness and decreased air entry in the right lower lung field. Laboratory studies showed CRP 190 mg/L, total/direct bilirubin 1.2/1.0 mg/dL, ALP 271 U/L, GGT 266 U/L, lipase 186 U/L, and ALT 29 U/L (Table 1). Chest radiography demonstrated a right‑sided loculated pleural effusion (Figure 1).

CT chest confirmed the loculated effusion and suggested that the biliary drain was irritating the pleura. Based on these findings, the drain was removed, and a diagnostic thoracocentesis was performed. Pleural fluid analysis revealed a bilirubin concentration of 1.251 mg/dL, exceeding the corresponding serum level of 0.9 mg/dL, immediately raising suspicion for biliopleural fistula. Despite these interventions, the effusion progressed, becoming loculated and resulting in near‑complete collapse of the right lower and middle lobes.

**Figure 2:**
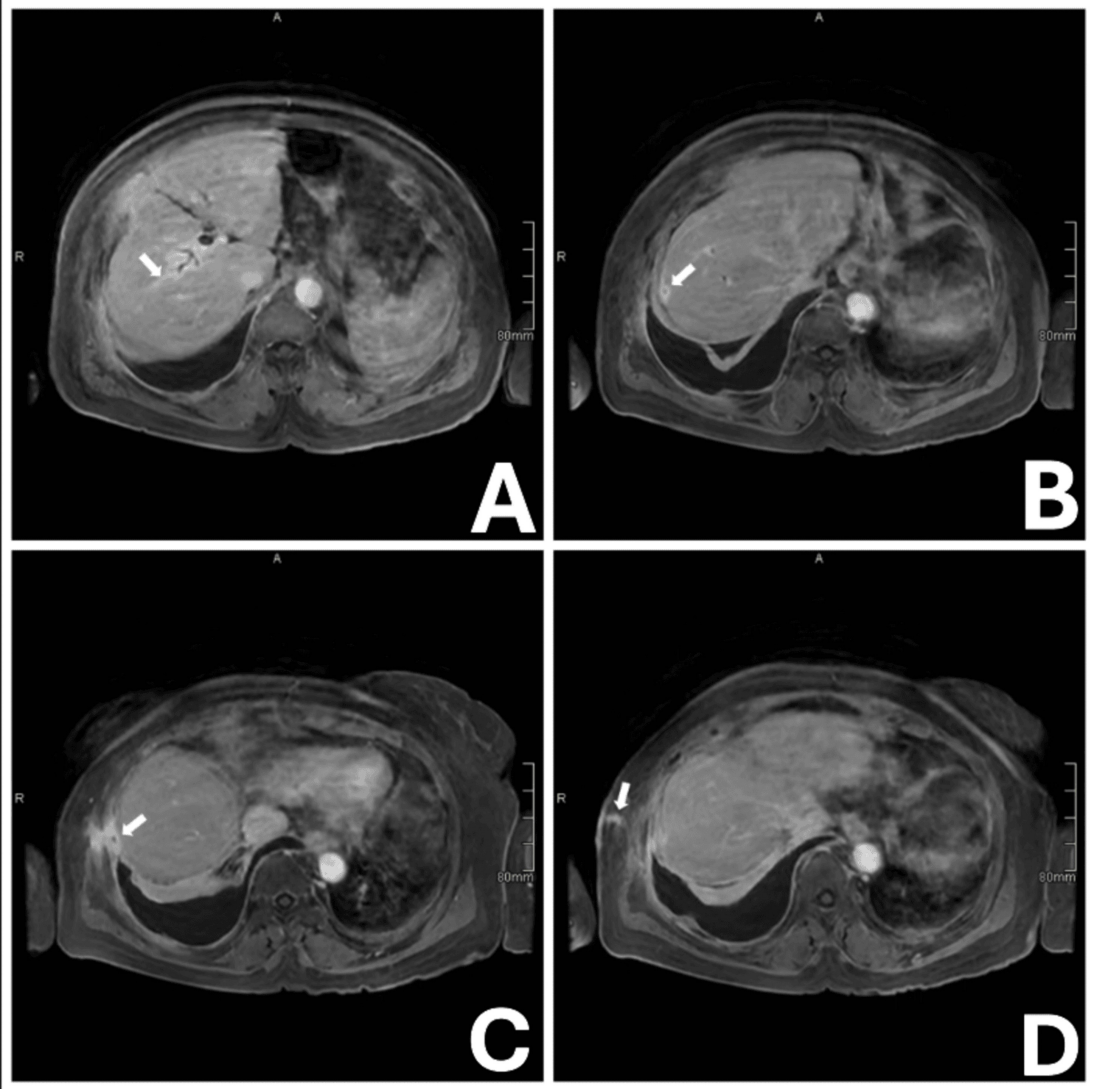
Magnetic resonance cholangiopancreatography (MRCP) demonstrating transpleural biliocutaneous tract. Sequential MRCP images (A-D) showing the transpleural tract extending from an intrahepatic biliary branch to the right costophrenic pleural space. The white arrow highlights the trajectory of the tract, which becomes progressively more conspicuous across panels A through D.

To further investigate the suspected biliopleural fistula indicated by thoracocentesis findings, MRCP was performed, and it demonstrated a tract from a right intrahepatic biliary branch traversing the hepatic capsule of segment VIII and communicating with the right costophrenic pleural space (Figure 2).

Given the persistence and progression of the loculated pleural effusion with suspicion of biliopleural fistula, a cardiothoracic surgery consultation was obtained. After a multidisciplinary team discussion, the decision was made to proceed with operative management. The patient subsequently underwent a right thoracotomy with decortication. Intraoperatively, bilious pleural fluid was encountered, confirming the diagnosis of biliopleural fistula. Her postoperative recovery was uneventful, and she was discharged in stable condition.

## Discussion

Biliopleural fistula is a rare but clinically significant complication of hepatobiliary disease, characterized by abnormal communication between the biliary system and pleural cavity. While most cases are linked to trauma, abscesses, or malignancy, iatrogenic causes (particularly following PTD) are increasingly reported in the setting of complex biliary obstruction and failed endoscopic access [5].

The pathophysiology involves bile leakage through a disrupted hepatic capsule and diaphragm, facilitated by elevated biliary pressure, procedural trauma, or infection‑induced necrosis [6]. Once bile enters the pleural cavity, it triggers an inflammatory cascade that may result in sterile or infected effusion, empyema, and respiratory compromise. The clinical presentation is often nonspecific, with dyspnea, pleuritic pain, fever, and cough mimicking pneumonia or pulmonary embolism [7]. This diagnostic overlap contributes to delayed recognition, underscoring the need for heightened suspicion in patients with recent hepatobiliary interventions who develop unexplained pleural effusions.

A key biochemical hallmark is pleural fluid bilirubin exceeding serum bilirubin, as demonstrated in our patient (pleural fluid bilirubin 1.251 mg/dL vs serum bilirubin 0.9 mg/dL), and diagnosis is confirmed with a pleural fluid-to-serum bilirubin ratio of >1 [8]. This finding strongly suggests biliary origin and should prompt further imaging [9]. MRCP is particularly valuable, offering high‑resolution visualization of subtle transdiaphragmatic tracts [3]. In our case, MRCP clearly delineated the fistulous communication, guiding definitive surgical management.

Management strategies vary depending on severity. Conservative measures, including external drainage, broad‑spectrum antibiotics, and supportive care, may suffice in stable patients with minimal leakage. However, surgical intervention is warranted in cases of loculated effusion, persistent biliary leakage, or respiratory compromise [10]. Thoracotomy with decortication remains the definitive treatment in complex or refractory cases, as in our patient who exhibited near‑complete collapse of the right lower and middle lobes [11].

This case adds to the limited literature on post‑PTD biliopleural fistula and highlights several important lessons. First, vigilance is required after PTD, particularly when patients re‑present with respiratory symptoms rather than abdominal complaints. Second, pleural fluid analysis is a simple yet powerful diagnostic tool, and bilirubin measurement should be considered in unexplained effusions following hepatobiliary procedures. Third, MRCP provides non‑invasive confirmation and guides surgical planning. Finally, early multidisciplinary coordination between gastroenterology, radiology, and thoracic surgery is essential to optimize outcomes [12].

Although this report describes a single case, it emphasizes the need for procedural awareness and timely recognition of rare complications. Broader documentation of similar cases will help refine diagnostic algorithms and management strategies for biliopleural fistula, ultimately improving patient safety in hepatobiliary interventions.

## Conclusions

Biliopleural fistula is a rare but serious complication of PTD, often presenting with nonspecific respiratory symptoms that delay diagnosis. This case illustrates the need for suspicion in patients with recent biliary interventions who develop unexplained pleural effusions. Pleural fluid analysis and MRCP are essential for diagnosis. Surgical intervention remains the cornerstone in cases complicated by loculated effusion or respiratory compromise. Early recognition and multidisciplinary coordination are critical for favorable outcomes.
